# The Layered Encapsulation of Vitamin B_2_ and β-Carotene in Multilayer Alginate/Chitosan Gel Microspheres: Improving the Bioaccessibility of Vitamin B_2_ and β-Carotene

**DOI:** 10.3390/foods11010020

**Published:** 2021-12-22

**Authors:** Peilong Liao, Shicheng Dai, Ziteng Lian, Xiaohong Tong, Sai Yang, Yashuang Chen, Weijie Qi, Xinhui Peng, Huan Wang, Lianzhou Jiang

**Affiliations:** 1 College of Food Science, Northeast Agricultural University, Harbin 150030, China; liaopeilong@neau.edu.cn (P.L.); 18443602143@163.com (S.D.); lianziteng@neau.edu.cn (Z.L.); tongxiaohong0110@163.com (X.T.); S201001027@neau.edu.cn (S.Y.); cys18337336195@163.com (Y.C.); 15725237855@189.cn (W.Q.); s201001904@neau.edu.cn (X.P.); jlzname@neau.edu.cn (L.J.); 2 Key Laboratory of Soybean Biology of Chinese Education Ministry, Harbin 150030, China; 3 School of Tea and Food Science & Technology, Anhui Agricultural University, Hefei 230036, China

**Keywords:** pH response, core-shell structure, simultaneous delivery, bioaccessibility

## Abstract

This research underlines the potential of alginate multilayered gel microspheres for the layered encapsulation and the simultaneous delivery of vitamin B_2_ (VB) and β-carotene (BC). Chitosan was used to improve the stability and controlled release ability of alginate-based gel microspheres. It was shown that a clear multilayered structure possessed the characteristics of pH response, and excellent thermal stability. The sodium alginate concentration and the number of layers had notable effects on mechanical properties and particle size of gel microspheres. Fourier-transform infrared spectroscopy and X-ray diffraction analyses further proved that VB and BC were encapsulated within the gel microspheres. Compared with the three-layer VB-loaded gel microspheres, the total release of VB from the three-layer VB and BC-loaded gel decreased from 93.23% to 85.58%. The total release of BC from the three-layer VB and BC-loaded gel increased from 66.11% to 69.24% compared with three-layer BC-loaded gel. The simultaneous encapsulation of VB and BC in multilayered gel microspheres can markedly improve their bioaccessibility and bioavailability. These results showed the multilayer gel microspheres synthesized herein have potential for applications in the layered encapsulation and simultaneous delivery of various bioactive substances to the intestinal tract.

## 1. Introduction

In recent years, several structured biopolymer-based matrices and carriers have already been developed to encapsulate and protect water-soluble or lipid-soluble bioactive substances [1,2,3,4]. Currently, various established delivery systems such as nanoparticles, microcapsules, multi-layer emulsion, and polymer-drug conjugates, are being used for the delivery of multiple bioactive substances [5,6,7,8].

Vitamins play an important role in the regulation of body functions. Vitamin B_2_ (VB), also known as riboflavin, is a water-soluble vitamin. β-carotene (BC), as an important member of the carotenoid family, which is a precursor substance of vitamin A. Both VB and BC are mainly absorbed in the colon, these substances have the ability to scavenge free radicals, improve vision, boost immunity, and protect skin health [9]. Indeed, Vitamins and carotenoids usually display a synergistic effects and have shown improvements in their functional properties for the human body when them were prescribed together [10,11]. A previous research showed that sufficient intake of both BC and VB could help prevent cognitive decline among elderly men with diabetes mellitus [12]. While VB is stable in acidic conditions, and it is destroyed under alkaline conditions, the opposite is true for BC [13]. Furthermore, the low solubility and bioavailability of VB and BC, limit their efficient transit and absorption in the human body [13]. Thus, there may be a major challenge associated with the development of delivery systems that can simultaneously encapsulate and deliver vitamin B_2_ and β-carotene on the same carriers.

The construction of gel-based delivery and a controlled release system had attracted much attention, as it simultaneously delivered various bioactive substances. Sodium alginate (SA) is an acidic polysaccharide rich in anions [14]. It has excellent biocompatibility, non-toxicity, and immunogenicity, owing to which it has been widely used for the delivery of bioactive substances [15,16,17,18]. However, there are still some limitations associated with the use of an alginate-gel based delivery system: alginate-based gel microspheres are highly porous and show a weak network, low adherence, and high burst release properties [19]. Fortunately, blending SA with other polymers is an effective method to circumvent these problems. Chitosan (CS), a type of cationic polysaccharide rich in amines, has been widely used in various pharmaceutical applications [20,21]. Previous research investigations have shown that chitosan not only improves the overall stability of the alginate beads, but also notably delays their release via the formation of electrostatic interactions [22,23,24]. Bajpai et al. [17] prepared calcium alginate/chitosan multilayered beads for delayed release of vitamin B_2_. Belščak et al. [25] used a latex/internal gel method to prepare gel microspheres that could simultaneously transport hydrophilic (from dandelion extract) polyphenols and β-carotene. Yadav et al. [26] reported that chitosan can fortify calcium alginate microspheres for the controlled delivery of dual drugs.

It has been speculated that the multilayered gel microspheres have the potential for layered encapsulation and protection for vitamin B_2_ and β-carotene from the external environment. To the best of our knowledge, thus far, no reports have investigated the simultaneous release behavior and bioavailability of VB and BC when they were layered encapsulated within multilayered gel microspheres. In this study, BC and VB were utilized as the model drugs and soybean protein isolate (SPI) was used as the aqueous phase. VB and BC were encapsulated within gel microspheres, which formed VB-in-gel layer-BC-in-gel layer systems. The swelling characteristics, particle size and mechanical properties, microstructure, and thermal stability of the prepared multilayered gel microspheres were evaluated. In addition, Fourier transform infrared spectroscopy (FT-IR) and X-ray diffraction (XRD) were used to analyze the interaction between SA and CS, and the encapsulation of VB and BC. The release behavior of VB and BC and the effects of multilayered structures on their bioavailability and bioaccessibility were further investigated via in vitro analyses of their release from the multilayer gel microspheres.

## 2. Materials and Methods

### 2.1. Materials

Chitosan (CS, level of deacetylation, 97%; ≥99.5% purity), Sodium alginate (SA, molecular weight in the range of 90–150 kDa; ≥97.7% purity), Calcium chloride (≥96.0% purity), β-carotene, BC (≥98% purity) and Vitamin B_2_, VB (≥97% purity) were bought from Yuanye Biotechnology Co., Ltd. (Shanghai, China). Soybean protein isolate (SPI, 91.6% protein) was purchased from DuPont Shineway Food Co., Ltd. (Luohe, China). The other chemicals were purchased from local shops in China.

### 2.2. Preparation of Sample

#### 2.2.1. Preparation of Chitosan-Alginate Core-Shell Gel Microspheres (L_1_)

The sodium alginate (SA) solution with varying concentrations (0.5%, 1%, 1.5%, 2.0%, and 2.5% *w*/*v*) were prepared by dispersing SA into distilled water; the pH value was adjusted to 5.0 using 1.0 M sodium hydroxide solution and hydrochloric acid solution. Then, CS powder was pre-dissolved in 1% acetic acid aqueous solution. The gelation medium consisted of 0.6% (*w*/*v*) CS and 5% (*w*/*v*) CaCl_2_, followed by adjusting the pH to 5.0. The SA solution was dropped into the gelation medium at room temperature with constant stirring and the flow rate was controlled at approximately 15 drops/min. The gel microspheres were allowed to stand in the gelation medium for 10 min and then removed. The microspheres were then washed with distilled water and dried at 35 °C until they reached constant weight. These gel microspheres shall henceforth be denoted as sample L_1_.

#### 2.2.2. Preparation of Multilayered Gel Microspheres (L_n_)

To prepare multilayered gel microspheres, the process of the sample L_1_ was repeated and the two-layer gel microspheres obtained. There were immediately put L_1_ into the SA solution for 20 min and then transferred into the gelation medium for 10 min. Then the gel microspheres were taken out and washed with distilled water, dried at 35 °C. These gel microspheres were named as sample L_2_. The three-layer gel microspheres were prepared by repeating the process above. These gel microspheres shall be denoted as sample L_3_.

#### 2.2.3. Preparation of VB-Loaded Gel Microspheres (L-VB)

The VB-loaded gel microspheres (containing VB in the core) were obtained by dropping the SA solution (pH 5.0) with vitamin B_2_ (0.25%, *w*/*v*) into the gelation medium. The one-layer VB-loaded gel microspheres shall be denoted as sample L_1_-VB. Then these microspheres were mixed repeatedly with the same SA solution to form multilayered gel microspheres. The two-layer and three-layer VB-loaded gel microspheres shall be denoted as sample L_2_-VB and L_3_-VB, respectively.

#### 2.2.4. Preparation of BC-Loaded Gel Microspheres (L-BC)

First, β-carotene was suspended in corn oil (0.5%, *w*/*v*). Then, an aqueous solution of 2.5% (*w*/*w*) (of proteins) SPI and corn oil (containing BC was emulsified (ULTRA-TURRAX, T18, IKA, Staufen, Germany) at 10,000 rpm/min for 3 min and homogenized at 60 MPa for three times to form an emulsion; next, the solution of SA was mixed with the emulsion at a ratio of 1:1 to form an emulsion-filled sol. The BC-loaded gel microspheres (containing BC in the core) were obtained by adding the emulsion-filled sol with BC (0.25%, *w*/*v*) into the gelation medium in a dropwise manner. The one-layer BC-loaded gel microspheres shall be denoted as sample L_1_-BC. Then these gel microspheres were mixed repeatedly with the SA solution to form multilayered gel microspheres. The two-layer and three-layer BC-loaded gel microspheres shall be denoted as sample L_2_-BC and L_3_-BC, respectively.

#### 2.2.5. Preparation of VB-BC-Loaded Gel Microspheres (L-VB-BC)

To prepare VB-BC-loaded gel microspheres (L_3_-VB-BC), the L_1_-VB gel microspheres were mixed with the emulsion-filled sol to form two-layer gel microspheres (L_2_-VB-BC), which were then transferred into the gelation medium for 10 min. These gel microspheres were mixed with the SA solution to form the three-layer gel microspheres gel microspheres (L_3_-VB-BC).

### 2.3. Swelling Study of Gel Microspheres

Briefly, the swelling property of the gel microspheres was measured according to the methods described by Sun et al. [27]. The pre-weighed gel microspheres were immersed in 0.1 M phosphate buffered solution (PBS) with pHs 2.0, and 7.4 at 37 °C, which served as the simulating fluid. The PBS was composed of disodium hydrogen phosphate and sodium dihydrogen phosphate and the pH was adjusted using 0.1 M HCl. At every 1-h interval, the swollen gel microspheres were weighed immediately after removing the excess droplets attached onto the surface of the gel microspheres using a filter paper. The weight change was measured for 6 h. The weight change was converted to a percentage using the formula shown below (1):

(1) $$\mathrm{Dynamic}\;\mathrm{weight}\;\mathrm{change}=\frac{\mathrm{Final}\;\mathrm{weight}-\mathrm{Initial}\;\mathrm{weight}}{\mathrm{Initial}\;\mathrm{weight}}\times 100\%$$

The measurements were repeated five times for all samples and the calculations were performed using the average data.

### 2.4. The Mechanical Properties of the Gel Microspheres

The fracture values (hardness and springiness) of the gel microspheres were measured using a TAXT plus analyzer apparatus (Stable Micro Systems, Godalming, UK) according to the method of Feng et al. [18]. The tests were performed on twenty gel microspheres using a P 35 cylindrical measurement probe. The trigger force and squeezing depth of the samples were set at 5 g and 10 mm at a rate of 1.0 mm/s, respectively. The compression of the gel microspheres was 40%. Each test was repeated three times. The hardness and springiness data were collected for range analysis and the optimal group of samples was selected.

### 2.5. Particle Size Measurement

The particle size of the gel microspheres was estimated as described by Ma et al. [28] with minor modifications. The particle size of the gel microspheres was measured previously using a digital caliper (0–150 mm, DL91150, Precisional Mesuring Tool, Deli Instruments, Ningbo, China). Thirty dried gel microspheres were selected and their particle size was measured with an accuracy of ±0.01 mm.

### 2.6. Differential Scanning Calorimetry (DSC) Analysis

The thermal stability of the gel microspheres was measured by using the method described by Fareez et al. [29]. About 10.0 mg of powders of gel microspheres (L_1_, L_2_, and L_3_) were placed in a ceramic crucible and DSC curves were constructed using a DSC instrument (DSC 3, Mettler Toledo, Greifensee, Switzerland). The analysis was performed at a temperature range of 20–200 °C at 10 °C/min and a dry flow of N_2_.

### 2.7. Analysis of the Microstructure of the Gel Microspheres

#### 2.7.1. Optical Microscopy (OM)

The morphology of the gel microspheres was observed using an optical microscope (BX53, Olympus Co. Ltd., Beijing China). Before observation, a thin layer of each gel microsphere (approximately 1 mm) was cut and placed on a glass slide.

#### 2.7.2. Confocal Laser Scanning Microscopy (CLSM)

Following the method of Lin et al. [30], each gel microsphere was cut into a thin layer (approximately 1 mm) and stained for 30 min. The staining solution was prepared by mixing Nile red (0.1%, *w*/*v*, in isopropyl alcohol) and Nile blue (0.1%, *w*/*v*, in isopropyl alcohol) at a ratio of 1:1. Confocal microscopy images were obtained using a Leica SP8 microscope (Leica Microsystems GmbH, Wetzlar, Germany), using excitation and emission wavelengths of 488 and 515 nm, respectively. 

#### 2.7.3. Scanning Electron Microscopy (SEM)

The morphology of the gel microspheres (PP3010T, Hitachi High-Technologies Corp., Tokyo, Japan) was obtained following the method described by Qin et al. [20], with slight modifications. Before observation, the gel microspheres were frozen, then the gel microspheres were sputter-coated with gold.

### 2.8. FT-IR Spectroscopy

The SA, CS, VB, BC, L_3_, 1.5% SA L_3_-VB-BC and 2.5% SA L_3_-VB-BC samples were studied using a Fourier transform infrared spectrophotometer (FT-IR; Thermo Fisher Technology Co., LTD, Shanghai, China). The FT-IR spectra were recorded on an FT-IR spectrophotometer, ranging from 4000 to 400 cm^−1^, using KBr pellets.

### 2.9. X-ray Diffraction (XRD) Analysis

The test conditions used for XRD analysis (Malvern Paralytical, Almelo, The Netherlands) were as follow: 2θ ranging from 5° to 70°; Cu was utilized as the light source. The generator voltage and tube current were set to 45 kV and 40 mA, respectively.

### 2.10. Release Studies 

#### 2.10.1. Drug Loading Capacity (LC) Measurement

To determine the drug loading capacity of the gel microspheres, a certain mass of the VB or BC-loaded gel microspheres (0.50 g) was dried and ground to a fine powder. The powder was then were placed in PBS (50 mL; pH 7.4). and shaken for 24 h at 30 °C in a water bath. After the drug was completely extracted from all the test gel microspheres, filtration was performed and the filtrate was reserved for later use.

The content of VB was measured by Bajpai and Tankhiwale’s [17] reported approach. Five milliliter aliquots of the filtrate were centrifuged at 2000× *g* for 5 min. The clear liquid in the middle layer was collected and then measured at 255 nm.

According to the methods described in Donhowe et al. and Roman et al. [31,32] reported to measure the content of BC, 5-mL aliquots of the filtrate were extracted with 5 mL of acetone: ethanol: hexane (25 mL:25 mL:50 mL, respectively), inverted 10 times and placed in capped test tubes. The top layer was collected and additional extractions were performed with 1 mL of n-hexane. After extraction, the content of BC was measured at 450 nm. The drug loading capacity was calculated as follows (2):

(2) $$\mathrm{LC}\;(\mathrm{mg}/g)=\frac{\mathrm{Total}\;\mathrm{bioactive}\left({\mathrm{vitamin}\;B}_{2}\;\mathrm{or}\;\beta -\mathrm{carotene}\right)}{\mathrm{Weight}\;\mathrm{of}\;\mathrm{bioactive}-\mathrm{loaded}\;\mathrm{gel}\;\mathrm{microspheres}}$$

#### 2.10.2. Drug Release Experiments

The release of the substances from the gel microspheres was analyzed according to the method described by Umaredkar et al. [33], with some modifications. The L_1_-VB, L_3_-VB, L_1_-BC, L_3_-BC and L_3_-VB-BC samples were performed in PBS (pH 2.0) for the first 2 h, then transferred to PBS with a pH 7.4 from 3 to 10 h. The bath parameters were set using an orbital shaking water bath as follows: 120 rpm/min, maintained at 37 ± 0.5 °C (SHA-B, Mycono Instruments Co., Ltd., Changzhou, China). At 1-h intervals, 5 mL aliquots were drawn and filtered every 1-h intervals using a pipette gun during a10 h period. The 5 mL of an equivalent fresh dissolution medium was added to maintain the same conditions after withdrawing each sample.

According to the method described in Section 2.10.1, the extent of release of VB or BC during digestion was evaluated as follows (3):

(3) $$\mathrm{Release}\;\left(\%\right)=\frac{\mathrm{Released}\;\mathrm{bioactive}\left({\mathrm{vitamin}\;B}_{2}\;\mathrm{or}\;\beta -\mathrm{carotene}\right)}{\mathrm{Total}\;\mathrm{bioactive}\left({\mathrm{vitamin}\;B}_{2}\;\mathrm{or}\;\beta -\mathrm{carotene}\right)}$$

#### 2.10.3. Bioaccessibility, Stability, and Bioavailability of VB and BC

The bioaccessibility, stability, and bioavailability of VB or BC were determined after simulated small intestinal digestion [34]. After in vitro simulated digestion, according to the method described in Section 2.10.1, the concentration of VB or BC was measured. The bioaccessibility, stability, and bioavailability of VB or BC were calculated as follows (4), (5) and (6), respectively, as shown below:

(4) $$\mathrm{Bioaccessibility}=\frac{C_{M}}{C_{D}}\times 100\%$$

(5) $$\mathrm{Stability}=\frac{C_{D}}{C_{I}}\times 100\%$$

(6) $$\mathrm{Bioavailability}=\frac{C_{M}}{C_{I}}\times 100\%$$

In these formulae C_I_, C_M_, and C_D_ are the concentrations of VB or BC added initially, in the micelle faction and in the total digesta at the end of the small intestinal digestion, respectively.

### 2.11. Statistical Analysis

All experiments were performed within 12 h of the preparation of samples prepared. Each test was performed at least twice in independent assays. Data analysis was performed using Origin 2019 (Origin Lab, Northampton, MA, USA). The results were analyzed by one-way analysis of variance (ANOVA) and Duncan’s multiple range test using the Statistica 20 software.

## 3. Results and Discussion

### 3.1. FT-IR Spectroscopy and XRD Analysis

**Figure 1:**
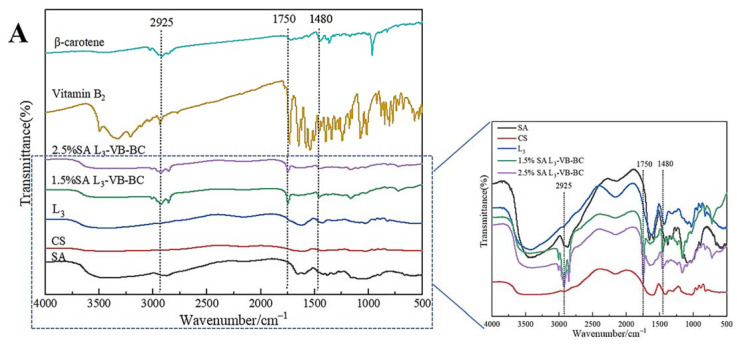

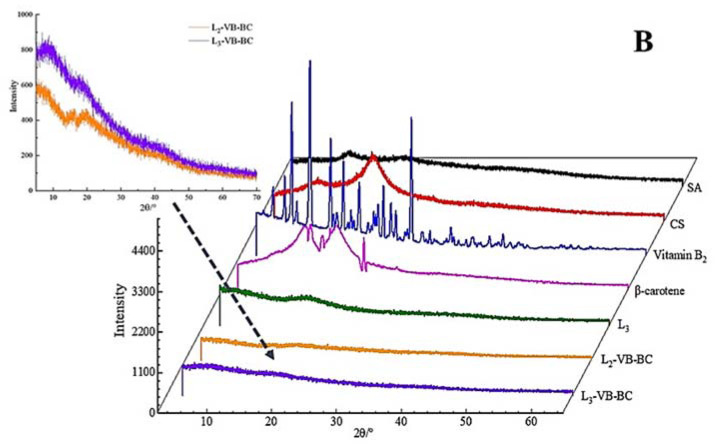
FT-IR and XRD of SA, CS, vitamin B_2_ (VB), β-carotene (BC) and gel microspheres. (**A**) FT-IR analysis of SA, CS, the three-layer-gel microspheres (L_3_), 1.5% SA VB and BC-loaded gel microspheres (1.5% SA L_3_-VB-BC), 2.5% SA VB and BC-loaded gel microspheres (2.5% SA L_3_-VB-BC), VB, and BC; (**B**) X-ray diffraction images of SA, CS, VB, BC, the three-layer-gel microspheres (L_3_), two-layer VB and BC-loaded gel microspheres (L_2_-VB-BC), and three-layer VB and BC-loaded gel microspheres (L_3_-VB-BC).

FT-IR spectroscopy is used to analyze the structural changes of substances. The FT-IR spectra of SA are shown in Figure 1A. The stretching vibrations of the O–H and C–H bonds are assigned at 3423.58 cm^−1^ and 2925.67 cm^−1^, respectively [35]. The peaks near 1437.23 cm^−1^ were caused by symmetric and asymmetric stretching vibrations of COO– groups. In Figure 1A CS, the peak at 3423.42 cm^−1^ is attributed to the O–H stretching vibrations. The bending vibration of N–H and the stretching vibration of C=O are observed at 1597.68 cm^−1^ and 1654.42 cm^−1^, respectively [35,36]. The peaks at 1258.15 cm^−1^ and 1064.33 cm^−1^ represent C–O stretching vibrations, which are common structures in CS.

The FT-IR spectroscopy of multilayered gel microspheres L_3_ differed markedly from the aforementioned observations, as shown in Figure 1. The peaks at 1597.68 cm^−1^ and 3423.42 cm^−1^ were shifted to 1617.45 cm^−1^ and 3470.21 cm^−1^, respectively, in case of the L_3_ microspheres. The asymmetric stretching vibrations of COO– groups changed from 1420.57 cm^−1^ to 1437.24 cm^−1^, which can be attributed to the cross-linking of Ca^2+^ ions with SA [37]; these changes indicated the cross-linking of Ca^2+^ ions and the polyelectrolyte complexation reaction of CS with SA [26]. The absorption peaks at 2925.57 cm^−1^ and 2854.43 cm^−1^ were caused by the vibration of the hydrophobic group CH_2_ in BC (Figure 1A β-carotene). 1746.41 cm^−1^ and 3009.11 cm^−1^ represent C–O stretching vibrations, which were common structures in VB (Figure 1A Vitamin B_2_). There were no significant changes in these peaks (Figure 1A L_3_-VB-BC), indicating that vitamin B_2_ and β-carotene were encapsulated within the gel microspheres.

In order to further prove that the vitamin B_2_ and β-carotene were encapsulated in the multilayer gel microspheres, CS, SA, the three-layer-gel microspheres (L_3_), VB, BC, VB and BC-loaded gel microspheres (L-VB-BC) were analyzed by XRD. For VB (Figure 1B), numbers of crystalline peaks were seen in the region of 10°–30°, suggesting VB had a clear crystal structure. The XRD pattern of BC (Figure 1B) shows there are strong peaks between 13° and 25°, indicating that BC has a highly crystalline structure [38]. While SA shows a typical smooth curve, the XRD patterns of CS did not accord with the result described by Ji et al. [24], in which CS showed strong peaks at 19.94° (Figure 1B CS), indicating the presence of different crystal morphologies. The three-layer-gel microspheres (L_3_) had a weak diffraction peak at 18.27°, suggesting it was significantly influenced by CS [39]. Compared to VB and BC, most of these peaks disappeared in the VB and BC-loaded gel microspheres, which was associated with low concentration of VB and BC. These changes were attributed to the increase in the proportions of molecules with higher degrees of freedom in the crystal structure, which can obscure the crystallinity of VB and BC [29]. The outcomes of XRD further confirm the dispersion of drugs into the gel microspheres.

### 3.2. CLSM of Gel Microspheres

**Figure 2:**
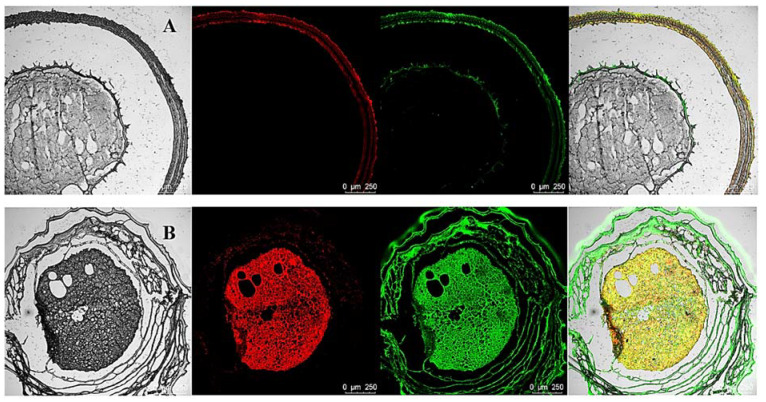
Confocal microscopy images of three-layer BC-loaded-gel microspheres (L_3_-BC) with embedding at different locations. Confocal microscopy images of 2.5% SA three-layer BC-loaded-gel microspheres (**A**). Confocal microscopy images of 1.5% SA three-layer BC-loaded-gel microspheres (**B**). The images were obtained by conventional optical microscopy and confocal fluorescence microscopy after sectioning the samples using a microtome.

The encapsulation of BC by the multilayered gel microspheres was investigated by CLSM analysis. The oil and protein were stained with Nile red and Nile blue, respectively (Figure 2). The gel microspheres show a notable core-shell structure, which is consistent with the structure observed in the microscopy analysis. Figure 2A shows that the proteins and oils are distributed in the shell layer. Interestingly, as shown in Figure 2B, both oils and proteins are distributed within the inside of the gel microspheres. These results indicate that bioactive substances can be layered encapsulated within multilayered gel microspheres. The multilayered structure confers the property of simultaneously carrying a variety of bioactive substances.

### 3.3. Mechanical Properties and Particle Size of the Gel Microspheres

It is known that the degree of deformation of gel microspheres under the action of applied external force is related to the particle size, number of layers, SA concentration and shell thickness [40], as shown in Table 1. The hardness and springiness of the gel microspheres increased with the concentration of SA and the increase in the number of layers, mainly due to the increase in the degree of cross-linking between Ca^2+^ and SA [41], which reflected that the higher the SA concentration, the denser is the shell structure. Thus, it may be inferred that the design of gel microspheres with different mechanical properties and physical properties can be achieved by varying the number of layers.

**Figure 3:**
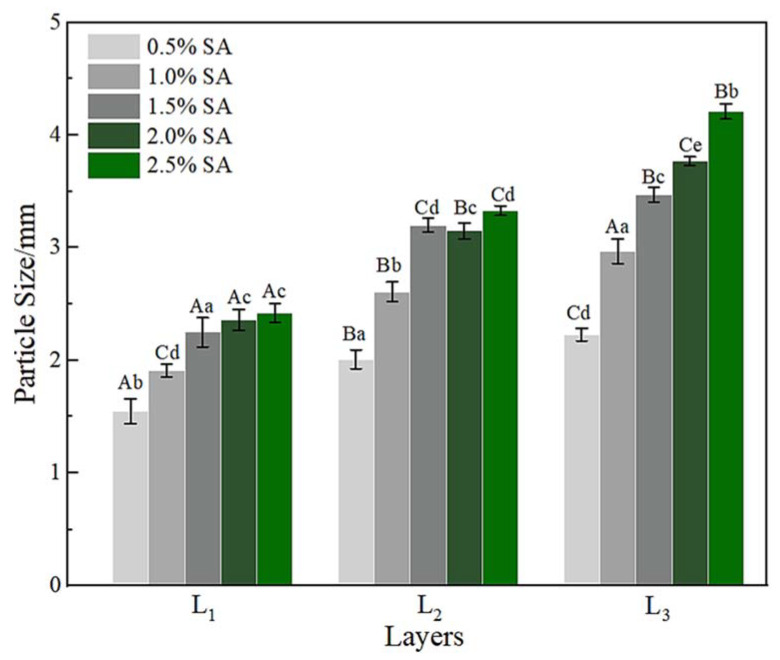
The particle size of L_1_, L_2_, and L_3_. L_1_: the one-layer-gel microspheres; L_2_: the two-layer-gel microspheres; L_3_: the three-layer-gel microspheres. Different lowercase letters indicate significant differences (*p* < 0.05) of the particle size within the same number of layers. Different capital letters indicate significant differences (*p* < 0.05) of the particle size between different number of layers.

The variation of the particle size of gel microspheres is shown in Figure 3. When the concentration of SA increased from 0.5% to 1.5%, the particle size of the one-layer gel microspheres (L_1_) increased significantly from 1.50 to 2.25 mm (*p* < 0.05). This was attributed to the fact that the shape of the gel microspheres formed was associated with the deformation of the droplet as it impacted the motionless gelation bath surface [37]. When the SA concentration was higher than 1.5%, this effect can be ignored, thus, the particle size tended to stabilize. The particle size of the multilayered gel microspheres increased markedly with an increase in the number of layers, which was consistent with the findings of a study by Anal et al. [42]. The maximum diameter of three-layer gel microspheres (L_3_) was 4.21 mm, while the minimum diameter was 2.23 mm. Higher the SA concentrations allowed for higher surface tension and thicker crusts, indicating that more SA remained on the multilayer gel microspheres as the SA concentration increased [43]. This further indicated the formation of a multi-layered structure.

### 3.4. DSC Analysis of the Gel Microspheres Measurement

**Figure 4:**
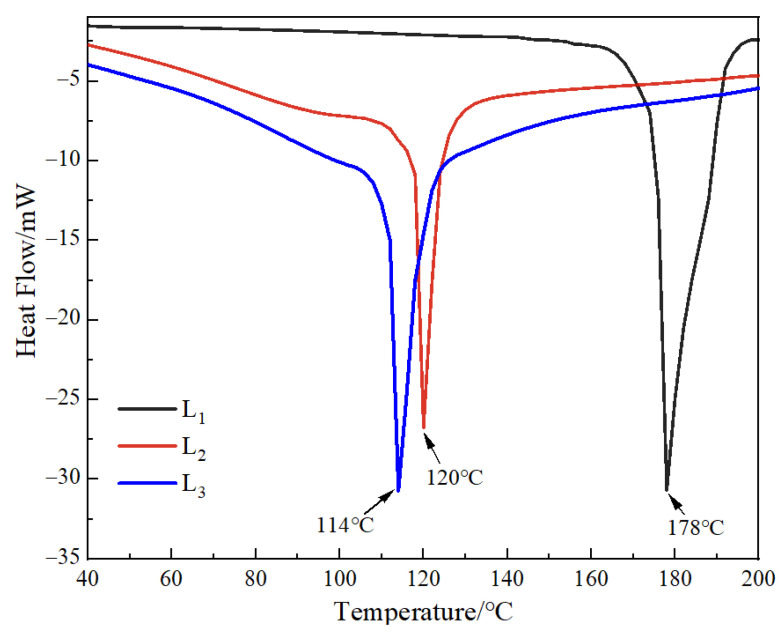
DSC thermograms of L_1_, L_2_, and L_3_. L_1_: the 2.5% SA one-layer-gel microspheres; L_2_: the 2.5% SA two-layer-gel microspheres; L_3_: the 2.5% SA three-layer-gel microspheres.

DSC can characterize the behavior of mixed phases in polymer blend systems, thus, demonstration of the thermal stability of the polymers can be ascertained (Figure 4) [44]. The DSC thermogram of the multilayered gel microspheres (L_1_, L_2_, and L_3_) showed that all the endothermic peaks occurred at more than 100 °C. It can also be anticipated that the other samples should also show excellent thermally stability below 100 °C. This reduction in the melting temperature of gel microspheres increased with an increase in the number of layers. This was related to the evaporation of water (both surface and crystallization water) in the gel microspheres [45,46], where more water molecules were evaporated with an increase in the number of layers. This study was focused on developing gel microspheres that can be used for oral drug delivery; it can demonstrate that the gel microspheres exhibit favorable stability of the body temperature (i.e., 37 °C).

### 3.5. Microstructure of Gel Microspheres

**Figure 5:**
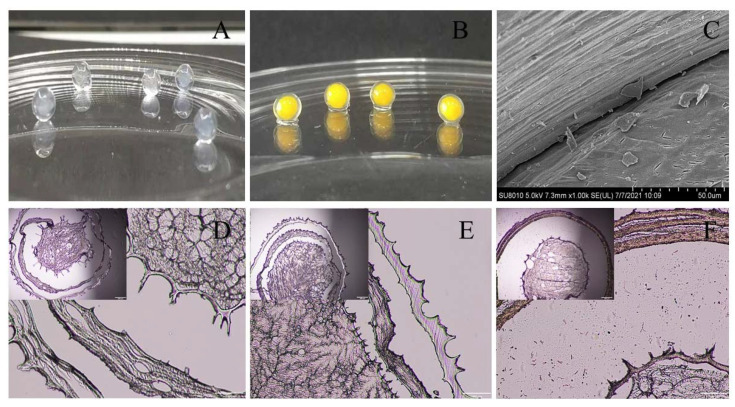
The digital photographs and microstructures of the gel microspheres. (**A**) Blank gel microspheres; (**B**) VB and BC-loaded-gel microspheres; (**C**) SEM photographs of cross sections of the microspheres; (**D**) Microscopic image of 0.5% SA three-layer-gel microspheres; (**E**) Microscopic image of 1.5% SA three-layer-gel microspheres; (**F**) Microscopic image of 2.5% SA three-layer-gel microspheres copy after sectioning the samples using a microtome.

The internal microstructure of the gel microspheres was observed using a microscope; these results are shown in Figure 5. The microspheres usually exhibit a spherical shell-like architecture with multiple layers inside each microsphere (Figure 5A,B). Therefore, the internal structure of the gel microspheres was further observed by scanning electron microscopy (SEM). The SEM image of multilayered gel microspheres (Figure 5C) shows that there are voids between the layers that can be used to preserve the drug, as described by Li et al. [43]. The optical microscopic image (Figure 5D) further shows that the gel microspheres had an obvious multi-layer structure. The multi-layer structure of the gel microspheres became denser and smoother as the concentration of SA increased. These findings may be related to the polycation properties of CS, which competed with Ca^2+^ to interact with the polyelectrolytes of SA [37]. When the concentration of SA increased, this competitive effect gradually diminished, leading to the formation of a denser shell. Lim et al. [47] reported that there was a loose gel structure of the gel microspheres, which showed weak gelation due to low concentrations of SA. Moreover, there were traces of tearing inside the gel microspheres; this may be because the internal structure of the gel microspheres was damaged to a certain extent during the sectioning process.

### 3.6. Swelling Properties of the Gel Microspheres

**Figure 6:**
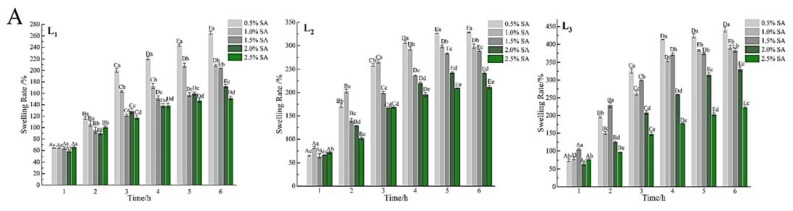

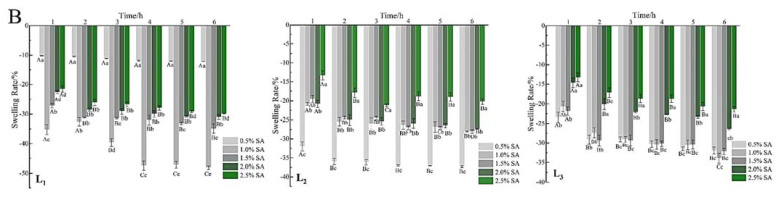
Swelling properties of the gel microspheres in phosphate-buffered solution with a pH of 7.4 (**A**) and 2.0 (**B**). L_1_: the one-layer-gel microspheres; L_2_: the two-layer-gel microspheres; L_3_: the three-layer-gel microspheres. Different lowercase letters indicate significant differences (*p* < 0.05) of the swelling degree within the same time period. Different capital letters indicate significant differences (*p* < 0.05) of the swelling degree between different time periods at the same pH.

The swelling behavior of the gel microspheres at pH 7.4, and 2.0 are shown in Figure 6. The percentage of swelling increased significantly (*p* < 0.05) with immersion time and the number of layers increased at pH 7.4. At approximately 4 h, the samples subjected to a pH of 7.4 were swollen to equilibrium and the swelling rate was more than 120% (Figure 6A). When the gel microspheres were placed in PBS, the swelling was mainly due to ion exchange between Ca^2+^ ions present in the “egg-box” cavity and Na^+^ ions [22]. Because the Ca^2+^ ions present in poly guluronate blocks units are exchanged with Na^+^ ions, and the carboxyl groups in SA electrostatically repel each other, thus the gel microspheres show swelling with the uptake of water [27,48].

Interestingly, the mass of gel microspheres decreased, and the microspheres shrank slowly (Figure 6B). SA (pKa is 3.5 at 37 °C) gained a positive charge when the pH of the solution was 2.0, which would inhibit the cross-linking of carboxyl groups with calcium; then some Ca^2+^ ions were dissociated and released from the gel microspheres [43]. In addition, the gel microspheres maintained good sphericity throughout the test at a low pH. This supported that the existence of electrostatic interactions between protonated –NH_3_^+^ groups and unionized –COOH groups under the acidic environments [49]. Thus, it may be concluded that the multilayered gel microspheres showed pH-responsive properties, which can remain stable in the harsh acidic environment of the stomach and protect the drugs from being destroyed.

### 3.7. In Vitro Release Studies of VB and BC from Gel Microspheres

**Figure 7:**
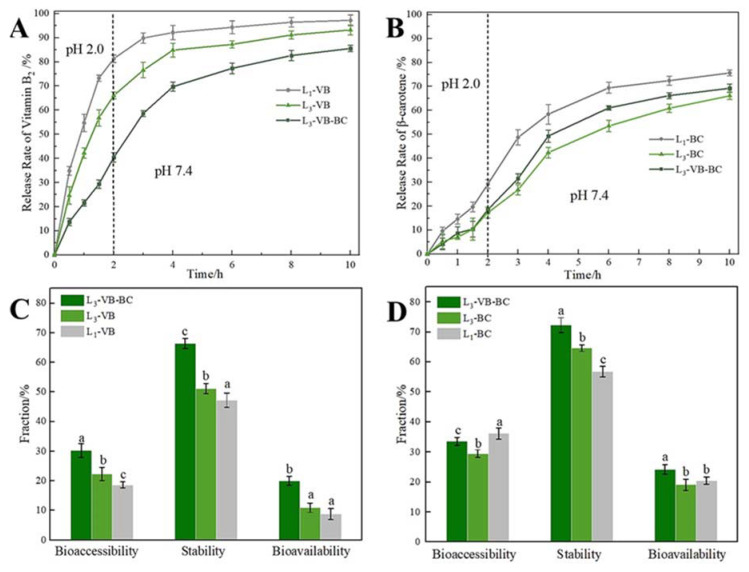
In vitro release studies of VB and BC from the gel microspheres. (**A**) The release profiles of VB from the gel microspheres; (**B**) The release profiles of BC from the gel microspheres; (**C**) Bioavailability, stability, and bioaccessibility of VB; (**D**) Bioavailability, stability, and bioaccessibility of BC. Different lowercase letters indicate significant differences (*p* < 0.05) of the same label.

According to the swelling characteristics, mechanical properties, and microstructure, the gel microspheres formed by 2.5% SA were submitted to study these conditions (e.g., single-layer-gel microspheres and multilayered gel microspheres), which could influence the loading capacity of the BC/VB-loaded gel microspheres as well as the release of VB and BC in different encapsulation layers in vitro digestion. Compared to the VB loading capacity of L_1_-VB (2.34 ± 1.51 mg/g), the VB loading capacity of L_3_-VB was reduced to 2.03 ± 0.89 mg/g. This was due to diffusion of VB into the gel medium during the incubation phase. In Figure 7A, nearly 70.0% of VB was released from the L_3_-VB and L_1_-VB after only 2 h. Then the release of VB tended to gradually stabilize after 6 h; then the rest of the drug was slowly released. The VB is unstable under alkaline conditions, thus the rapid release of VB in the stomach, once it moves to the alkaline conditions of colon, which can quickly be absorbed. Release was divided into two main methods: drug release via degradation of the alginate network and drug diffusion through the alginate network for the drug release mechanism of the gel microspheres system [50]. When the gel microspheres were placed at pH 2.0, the COO– groups of SA started to protonate, forming uncharged –COOH groups; thus, the alginate network became loose due to reduction in the degree of crosslinking of SA with Ca^2+^, and the electrostatic repulsion between the SA and CS increased [49]. Being water-soluble, VB quickly diffused into the solution through these loose network structures, leading to the burst release of VB. However, these release behaviors indicated that multilayer gel microspheres significantly retarded the release of VB as the number of layers increased. This phenomenon may be due to the network structure of the alginate gel; this structure may have limited the release of the drug [51].

There was no notable difference for the BC loading capacity of L_1_-BC (2.92 ± 1.13 mg/g) and L_3_-BC (2.88 ± 0.72 mg/g). Interestingly, the release behavior of BC was different from that for VB. BC-loaded gel microspheres (Figure 7B) showed a sustained slow-release mode at pH 2.0, with less than 20% of BC being released within 2 h. This was due to the role of the outermost SA layer, which served as a diffusion barrier [22]. When BC-loaded gel microspheres were transferred into the artificial intestinal fluid (pH 7.4), they rapidly released the drug for nearly 4 h. When acidic/treated gel microspheres were transferred to the alkaline environment, the gel microspheres swelled faster but did not show higher water absorption values, which was related to loosening of their binding structures [52]. The protein was filled in a gel network that can help the gel microspheres absorb water faster [53]. Finally, the gel microspheres began to disintegrate along with the rapid release of BC.

For L_3_-VB-BC, the VB and BC loading capacity were 2.79 ± 1.25 and 2.73 ± 1.10 mg/g, respectively. The enhanced VB loading capacity was related to the fact that the outer emulsion-filled gel layer can reduce the loss of VB. Moreover, the amount of VB released from L_3_-VB-BC was lower than that from L_3_-VB. The release of BC from gel microspheres was limited because of the hydrophobic of BC and its contact with the gel layer [18]. However, the release of BC from L_3_-VB-BC was higher than that from L_3_-BC, indicating that the dissolution of VB promoted the release of BC. An increase and decrease in the cumulative release of BC (69.24%) and VB (85.58%) were observed. This suggested that multilayered gel microspheres demonstrate the efficient delivery of bioactive substances in the colon, enabling the enhanced release of VB and BC in the intestinal tract.

Bioavailability and bioaccessibility can be used to evaluate the delivery efficiency of delivery systems [54]. In Figure 7C, the bioaccessibility and stability of VB are low for L_1_-VB. There are two main reasons for that: firstly, VB readily dissolves in alkaline solutions [13]; and secondly, the VB dissolves quickly through these loose gel network structures. The bioavailability and stability of VB increased notably with the increase in the number of layers; this was related to the increase in the time required for its diffusion into the medium. The bioaccessibility of BC decreased with the numbers of layers, however, its stability increased significantly (Figure 7D). This was attributed to the gel layer and the presence of oil, which limited the release of BC. In addition, a certain amount of the BC was still encapsulated in the undigested lipid droplets [34]. For L_3_-VB-BC, the bioaccessibility, stability and bioavailability of VB and BC were significantly improved (Figure 7C,D). This result indicated that the release of VB can improve the bioaccessibility of BC, and the release of BC also can improve the stability of VB. It also predicted that released BC was possibly limited by SA, CS and the formation of an emulsion in the solution medium; this may have contributed towards an increase in the stability of VB.

In general, the sustained release of VB or BC from multilayered gel microspheres was excellent; however, the low bioaccessibility and bioavailability of the encapsulated substances limited their delivery drugs into the gastrointestinal tract. The simultaneous encapsulation of VB and BC within multilayered gel microspheres notably improved their bioaccessibility and bioavailability. Considering that food needs to pass through the oral region to the gastric fluid and finally reach the intestine for digestion or absorption, the multilayer gel microspheres have the potential to simultaneously encapsulate and deliver multiple bioactive substances to the small intestine in these variable environments.

## 4. Conclusions

The results of FT-IR and XRD analysis confirmed the electrostatic interaction between CS and SA, and the VB and BC were encapsulated within the gel microspheres. The mechanical properties and particle size of these multilayered gel microspheres were controlled by the number of layers and the concentration of SA. The prepared multilayered gel microspheres were spherical and their clear core-shell structure was seen in the microscopic images. The swelling results exhibited remarkably diverse responses of multilayered gel microspheres to external conditions (pH 7.4, and 2.0). The multilayered gel microspheres showed more delay in the release of VB and BC, compared with the one-layer VB-loaded gel microspheres or one-layer BC-loaded gel. The release behavior of VB and BC-loaded gel microspheres suggested that released BC was possibly restricted by SA, CS and the formation of the emulsion, which improved stability of VB. The simultaneous encapsulation of VB and BC within multilayered gel microspheres notably improved their bioaccessibility and bioavailability. The above results indicated that the multilayered gel microspheres had the ability to simultaneously encapsulate water-soluble and lipid-soluble bioactive substances, enabling the delivery of these substances into the gastrointestinal tract. Thus, the multilayered gel microspheres show great potential as delivery systems for improving the digestion and absorption of multilayer-gel-encapsulated nutrients in the body.

## Figures and Tables

**Table 1:** Springiness and hardness of gel microspheres.

Sample	L_1_		L_2_		L_3_	
	Hardness(g)	Springiness	Hardness(g)	Springiness	Hardness(g)	Springiness
0.5% SA	54.04 ± 0.49 ^e^	0.607 ± 0.005 ^d^	68.81 ± 0.25 ^e^	0.749 ± 0.012 ^d^	183.62 ± 0.58 ^e^	0.79 ± 0.004 ^d^
1.0% SA	59.90 ± 0.44 ^d^	0.712 ± 0.006 ^c^	74.34 ± 0.92 ^d^	0.772 ± 0.004 ^c^	368.56 ± 1.88 ^d^	0.866 ± 0.0042 ^c^
1.5% SA	65.15 ± 0.22 ^c^	0.787 ± 0.006 ^b^	112.13 ± 0.27 ^c^	0.796 ± 0.007 ^b^	457.14 ± 1.20 ^c^	0.923 ± 0.006 ^b^
2.0% SA	86.76 ± 0.34 ^b^	0.833 ± 0.03 ^a^	164.73 ± 0.71 ^b^	0.885 ± 0.003 ^a^	589.96 ± 1.67 ^b^	0.968 ± 0.004 ^a^
2.5% SA	135.61 ± 0.46 ^a^	0.852 ± 0.13 ^a^	442.75 ± 1.07 ^a^	0.891 ± 0.052 ^a^	632.12 ± 0.04 ^a^	0.959 ± 0.008 ^a^

L_1_: the one-layer-gel microspheres; L_2_: the two-layer-gel microspheres; L_3_: the three-layer-gel microspheres. Values with different superscript letters are significantly different (*p* < 0.05).
